# Widespread remodeling of mid-coding sequence nucleosomes by Isw1

**DOI:** 10.1186/gb-2010-11-5-r49

**Published:** 2010-05-10

**Authors:** Itay Tirosh, Nadejda Sigal, Naama Barkai

**Affiliations:** 1 Department of Molecular genetics, Weizmann Institute of Science, Herzl street, Rehovot 76100, Israel; 2 Current address: Department of Molecular Microbiology and Biotechnology, George S. Wise Faculty of Life Sciences, Tel-Aviv University, Ramat Aviv, Tel-Aviv 69978, Israel

## Abstract

**Background:**

The positions of nucleosomes along eukaryotic DNA are defined by the local DNA sequence and are further tuned by the activity of chromatin remodelers. While the genome-wide effect of most remodelers has not been described, recent studies in *Saccharomyces cerevisiae *have shown that Isw2 prevents ectopic expression of anti-sense and suppressed transcripts at gene ends.

**Results:**

We examined the genome-wide function of the Isw2 homologue, Isw1, by mapping nucleosome positioning in *S. cerevisiae *and *Saccharomyces paradoxus *strains deleted of ISW1. We found that Isw1 functions primarily within coding regions of genes, consistent with its putative role in transcription elongation. Upon deletion of ISW1, mid-coding nucleosomes were shifted upstream (towards the 5' ends) in about half of the genes. Isw1-dependent shifts were correlated with trimethylation of H3K79 and were enriched at genes with internal cryptic initiation sites.

**Conclusions:**

Our results suggest a division of labor between Isw1 and Isw2, whereby Isw2 maintains repressive chromatin structure at gene ends while Isw1 has a similar function at mid-coding regions. The differential specificity of the two remodelers may be specified through interactions with particular histone marks.

## Abstract 2

In yeast, the chromatin remodeler Isw1 shifts nucleosomes from mid-coding, to more 5’ regions of genes and may regulate transcriptional elongation.

## Background

Chromatin is composed of core nucleosome particles, each containing approximately 147 bp of double-stranded DNA wrapped around a histone octamer [1]. Nucleosomes restrict the accessibility of proteins to the DNA, thereby influencing DNA transcription, replication, recombination and repair [2-4]. Nucleosome positioning is determined, to a large extent, by the local DNA sequence and its affinity to nucleosomes [5-7], but is also dynamically altered by the activity of a large number of chromatin-associated proteins [8,9]. Transcription factors and other DNA-binding proteins can influence nucleosome positioning by competing with nucleosomes for binding to DNA [5,10]. In addition, chromatin regulators directly modify the positions, or the states, of nucleosomes.

Chromatin regulators are classified into three main categories: histone variants, chromatin modifiers and chromatin remodelers. Of these, chromatin remodelers directly alter the histone-DNA contacts and are expected to have the strongest influence on nucleosome positioning [11]. Chromatin remodelers fall into four main families (*SWI/SNF*, *ISW1*, *CHD *and *INO80*) that are characterized by different domains and biological functions. The functions of these remodelers have been studied extensively using single genes and *in vitro *systems, but their effects on the genome-wide positions of nucleosomes have been mapped for only a few remodelers [12-14]. Recent genome-wide mapping of nucleosome positioning in a strain deleted of *ISW2 *revealed that Isw2 shifts the positions of nucleosomes around transcription initiation and transcription termination sites, thereby preventing transcription from antisense and suppressed sites [15]. The homologous protein Isw1 was also shown to alter nucleosome positioning at particular loci [15], but its genome-wide role, and in particular how it differs from that of Isw2, were not described. Interestingly, Isw1 was shown to form two distinct complexes (Isw1a and Isw1b) that appear to play roles in transcription initiation and elongation, respectively [15-17].

Here, we describe the genome-wide influence of Isw1 on nucleosome positioning. *ISW1 *deletion preferentially influenced nucleosome positioning within coding regions, and in particular shifted the positions of nucleosomes at mid-coding regions towards the 5' end of the genes. Our data suggest a 'division of labor' between Isw1 and Isw2, specified through distinct histone modifications, and implicates Isw1 in transcriptional elongation and in preventing cryptic initiation within genes.

## Results

**Figure 1:**
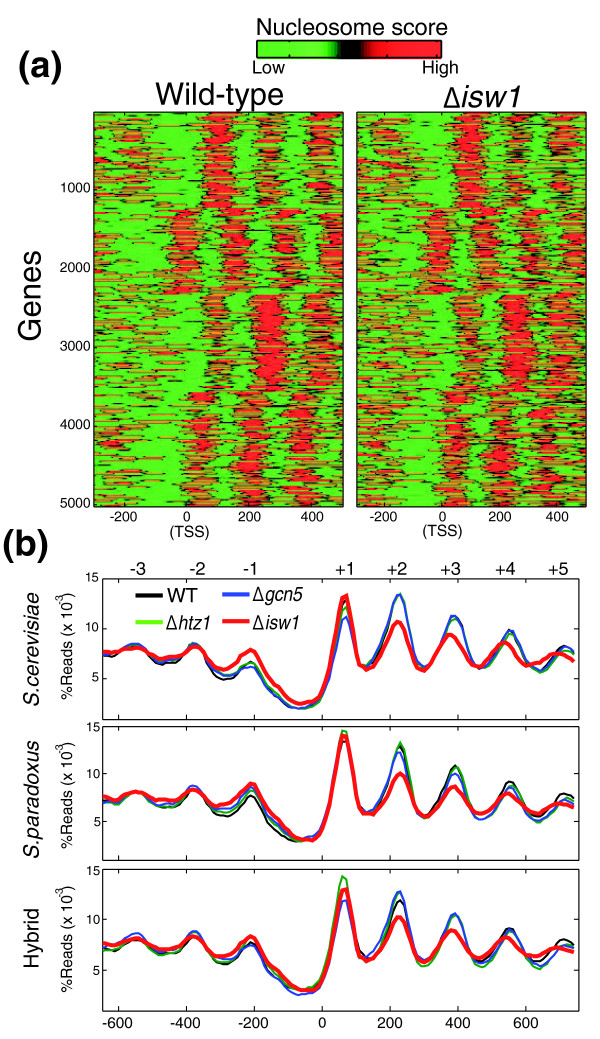
**Global patterns of nucleosome positioning in wild-type and deletion mutant strains**. **(a) **Heatmaps of nucleosome scores for the wild-type and *Δisw1 S. cerevisiae *strains. Genes were divided into four clusters by k-means clustering. **(b) **Average pattern of nucleosome positioning for all yeast genes in the wild-type (WT) and three mutant strains, shown as percentage of reads mapped to different positions relative to transcription start sites (TSSs). Nucleosome numbering is shown at the top [52]. The same analysis was performed for *S. cerevisiae *(top), *S. paradoxus *(middle) and their inter-specific hybrid (bottom), using the TSS positions from *S. cerevisiae *[53].

We used Illumina high-throughput sequencing to map genome-wide nucleosome positioning in wild-type yeasts and in mutants deleted of *ISW1 *(Figure 1a). Experiments were performed in duplicates, for *Saccharomyces cerevisiae*, for its close relative *Saccharomyces paradoxus *[18], and for the inter-specific hybrid obtained by mating these two species. Samples from the two species were pooled and sequenced together, and reads were mapped to either one of the genomes, thus excluding the analysis of highly conserved genomic regions (approximately 13% of the genome; see Materials and methods). An inter-species analysis and the evolutionary implications will be presented elsewhere while here we focus on the influence of deleting *ISW1*, which is largely conserved between the two species and observed also in the hybrid. As additional controls, we profiled mutants deleted of *HTZ1*, a histone variant associated primarily with the -1 and +1 nucleosomes that was shown recently to exert only minor effects on nucleosome positioning [14,19,20], and *GCN5*, a histone modifier (acetyl-tranferase). Gcn5 does not alter nucleosome positioning directly, but modulates histone acetylation (and thus charge), which is expected to have some influence on nucleosome positioning [21-23].

We began by comparing the typical patterns of nucleosome positioning surrounding the transcription start site (TSS), as observed when aligning all genes with respect to the TSS and averaging over all genes (Figure 1b). As shown in previous studies [24,25], in a wild-type strain this average pattern consists of a promoter region that is relatively depleted of nucleosomes (nucleosome-free region) followed by an array of well-phased nucleosomes with gradually decreasing occupancy at the coding region. We found the exact same pattern also in the control strains deleted of *HTZ1 *or *GCN5*. The average nucleosome profile of the *ISW1 *deleted cells, however, deviated significantly from this pattern, displaying decreased occupancy of nucleosomes within the coding region. This reduced occupancy at coding regions was observed in both species and also in the hybrid (Figure 1b).

We asked whether the genes directly bound by Isw1, as determined by chromatin immunoprecipitation (ChIP) [26], are more sensitive to its deletion compared to other genes (Figure S3 in Additional file 1). Such correlations were observed for the two control strains, *Δhtz1 *[27] and *Δgcn5 *[23], where the deletion affected bound genes significantly more than unbound genes. In contrast, there was only a slight difference between genes detected as bound or unbound by Isw1 or by the Isw1-binding proteins Ioc2 and Ioc3. These results may suggest that remodeling requires only transient binding of Isw1 to nucleosomes, interactions that are difficult to detect using current binding assays done with wild-type Isw1 (as was indeed demonstrated for Isw2 [28]). Furthermore, Isw1 binding was examined only for promoter regions [26], while our data suggest that Isw1 exerts a more significant effect within coding regions [16,17].

**Figure 2:**
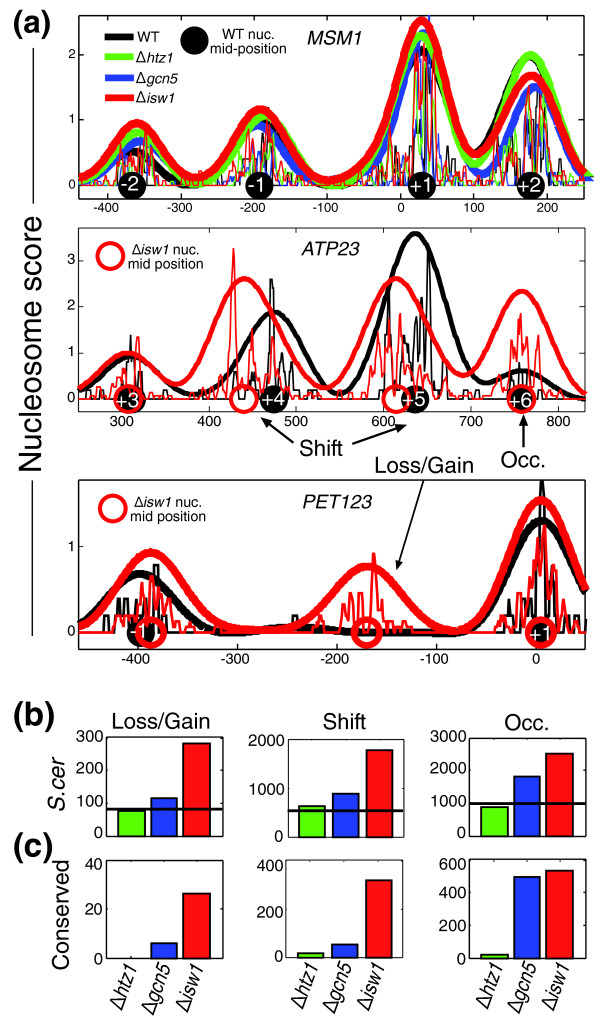
**Remodeling of individual nucleosomes**. **(a) **Read density and calculated nucleosome scores for wild-type (WT) and three mutant strains at three genes (*MSM1*, which has similar nucleosome patterns for all strains, and *ATP23 *and *PET123*, which have different nucleosome patterns at *Δisw1*), including examples of the three classes of changes that we defined: shift, loss/gain and occupancy (Occ.). Estimated nucleosome center positions are indicated as black (wild-type) or red (*Δisw1*) circles with nucleosome numbering. **(b) **Number of changes identified over all genes examined. Horizontal lines indicate the number of changes observed among biological repeats. **(c) **Number of changes that are found in both *S. cerevisiae *and *S. paradoxus*.

We next searched for particular nucleosomes whose positions or occupancies were altered in the deletion mutants (Additional file 2). For each gene, we compared the density of nucleosome reads and the smoothed profile (nucleosome scores) between the wild type and mutant strains and defined three classes of differences (Figure 2a; Materials and methods): nucleosomes whose occupancies are altered by at least two-fold (Occ.); nucleosomes whose positions are changed significantly by at least 15 bp (Shift) and nucleosomes that are present in one strain but absent in another (Loss/Gain). To estimate the number of changes that would be observed by chance, we performed similar analyses comparing the biological repeats performed for each of the mutant strains.

The number of changes in *Δhtz1*, relative to wild-type, was similar to that found between biological repeats (Figure 2b). Moreover, very few changes at *Δhtz1 *were conserved among the two different species (Figure 2c). Consistent with previous studies [14], these results suggest that Htz1 has little influence on nucleosome positioning and that the observed differences at Htz1-bound genes are subtle. More changes were obtained in *Δgcn5*, but these were typically small. In contrast, the number of changes in *Δisw1 *was considerably higher than that found between biological repeats, with many changes conserved among the species (Figure 2b, c).

### Isw1 nucleosome remodeling at coding-regions

**Figure 3:**
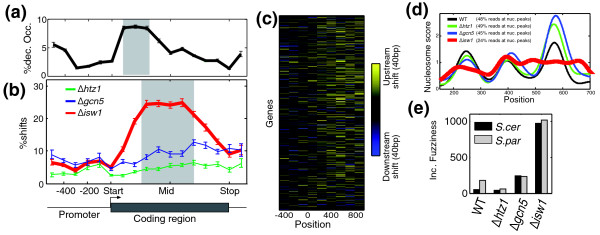
**Nucleosome remodeling by Isw1**. **(a) **Percentage of nucleosomes with at least two-fold reduced occupancy upon deletion of *ISW1*, as a function of their normalized location with respect to the start and stop codons. Shaded area shows the strongest enrichment. **(b) **Percentage of nucleosomes with shifts (>15 bp) upon deletion of the three chromatin regulators, as a function of their normalized location with respect to the gene start and stop codons. **(c) **Heatmap of nucleosome shifts across approximately 5,000 *S. cerevisiae *genes, sorted by transcription rates [54] (top, lowly transcribed; bottom, highly transcribed). See Figure S2 in Additional file 1 for similar heatmaps of the other mutant strains and for heatmaps of changes in nucleosome occupancy. **(d) **Increased nucleosome fuzziness at the coding region of *HOL1 *in *Δisw1 *cells. Shown are *HOL1 *nucleosome scores for all strains, and the percentage of reads that map to within 20 bp of estimated nucleosome center positions is indicated for each strain. **(e) **Number of genes with increased fuzziness for each strain of *S. cerevisiae *(black) and *S. paradoxus *(grey). Genes were defined to have increased fuzziness in a particular strain if the percentage of reads that map to within 20 bp of the estimated nucleosome center positions was lower by at least 5% than that of all other strains, while the number of predicted nucleosomes is unchanged. WT, wild type.

Thus, consistent with its role as a chromatin remodeler, deletion of *ISW1 *led to extensive changes in nucleosome positioning and occupancy. Notably, these effects were primarily within coding regions (Figure 3). First, most of the changes in nucleosome occupancy observed upon deletion of *ISW1 *were localized at nucleosomes +2 to +4 within the coding regions, and typically reduced nucleosome occupancy at this region (Figure 3a). Second, the positioning of nucleosomes at coding regions, but not at intergenic regions, became fuzzier upon deletion of *ISW1 *(Figure 3d, e). For example, only 25% of the reads at the *HOL1 *coding region mapped to within 20 bp of the estimated nucleosome positions in the *Δisw1 *strain, compared to 45 to 49% of the reads in each of the other strains (Figure 3d). Fuzziness increased in the *Δisw1 *strain for approximately 1,000 genes (Figure 3e), whereas decreased fuzziness was observed for only 44 genes (Figure S4 in Additional file 1). Similar results were obtained for *S. paradoxus *(Figure 3e) and for the hybrid (not shown).

Third, shifts of nucleosome positions were particularly enriched at the mid-coding region of genes (Figure 3b, c). Notably, the shifted positions, as observed in the *Δisw1 *strain, were typically more consistent with sequence-based predictions than the positions observed in the wild-type strain (Figure S5 in Additional file 1). This indicates that Isw1 normally slides nucleosomes into energetically less-favorable positions. Thus, the observed shifts most likely reflect the direct ATP-dependent remodeling activity of Isw1 [29,30], although we cannot exclude the possibility that some of these changes are due to indirect effects. We therefore focused our subsequent analysis on Isw1-dependent shifts in nucleosome positions. These shifts are widespread and are comparable in magnitude to those found upon RNA polymerase (PolII) inactivation (see below).

In principle, the enrichment of Isw1-dependent shifts at mid-coding regions could be explained by statistical positioning: if nucleosome positions are primarily determined by border elements positioned at the two ends of the coding region, then nucleosomes at the middle of genes, where shifts in *Δisw1 *are mostly observed, would be less constrained and more susceptible to regulation [31-33]. However, as described below, the patterns of Isw1-dependent shifts argue against this interpretation and instead support an active mechanism that directs Isw1 activity to mid-coding regions.

**Figure 4:**
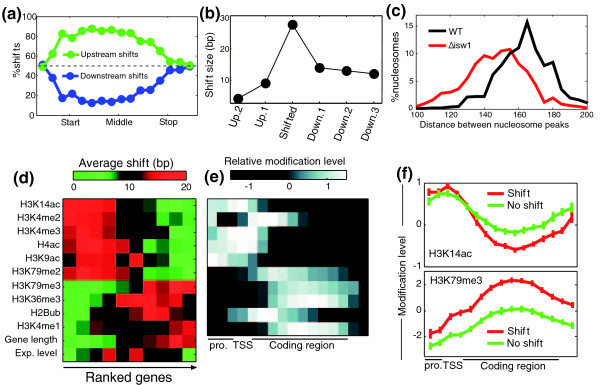
**Patterns of Isw1-dependent shifts**. **(a) **Percentage of Isw1-shifts that are upstream (green; nucleosomes are moved towards the 5' end in *Δisw1*) and those that are downstream (blue; nucleosomes are moved towards the 3' end in *Δisw1*) as a function of the relative position within genes (with respect to the start and stop codons). **(b) **Asymmetric effects at nucleosomes adjacent to those with shifts. For each gene with Isw1-shifts above 15 bp, we examined the extent of upstream shifts at the maximally shifted nucleosome and at its upstream and downstream adjacent nucleosomes, and the average shift sizes are shown. **(c) **Distribution of estimated distances between nucleosome centers of mid-coding nucleosomes (that are shifted upstream in *Δisw1*) and their flanking upstream nucleosomes, for wild-type (WT) and *Δisw1 *strains. **(d) **Average sizes of Isw1 upstream shifts at the +5 nucleosome for 10 subsets of genes ordered by various histone modifications, gene length, or mRNA expression levels. H3K79me2/3 and H2Bub were taken from Schulze *et al*. [55] and all other modifications from Pokholok *et al*. [56]. **(e) **Average levels of the histone modifications in (d), normalized to mean of zero and standard deviation one, throughout promoters and coding regions. **(f) **Average patterns of modifications for genes with Isw1 upstream shift of the +5 nucleosomes of at least 20 bp (red) and those without upstream shifts (green), shown for H3K14 acetylation (top) and H3K79 trimethylation (bottom).

First, the presence of Isw1-dependent shifts at mid-coding regions is not correlated with the presence of nucleosome-free regions, or with strong positioning sequences at the ends of genes [31] (not shown). Second, these shifts display a strong direction bias: almost exclusively, the shifts occur in the direction opposite to that of elongation (Figure 4a) - in 85% of the cases, mid-coding nucleosomes were shifted upstream in *Δisw1*, towards the start codon. This highly significant directionality (*P *< 10^-16^) is not expected by models of statistical positioning, but suggests instead that Isw1-dependent shifts reflect its function during elongation [15]. Third, although the shifts propagate to flanking nucleosomes, as expected from statistical positioning models, this propagation is again biased, with downstream nucleosomes affected significantly more than upstream nucleosomes (Figure 4b). For example, the +4 nucleosome of *ATP23 *is shifted upstream by 34 bp, its downstream nucleosome (+5) is shifted by 22 bp, but its upstream nucleosome (+3) is not shifted at all (Figure 2a). As a result, the linker region between the +3 and +4 nucleosomes is practically abolished. More generally, the distance between the predicted centers of the Isw1-shifted nucleosomes and their upstream flanking nucleosomes drops from a median of 165 bp in the wild type to only 150 bp in *Δisw1 *(Figure 4c). Given the expected nucleosome length of 147 bp, this suggests that there are virtually no linker regions between these nucleosome pairs in *Δisw1*.

### Isw1 remodeling is correlated with H3K79me3

How is the specificity of Isw1 to mid-coding nucleosomes of particular genes established? Previous studies have shown that chromatin remodelers, including Isw1 and Isw2, interact with histone modifications, suggesting that Isw1 might be recruited through specific interactions with histone marks that characterize mid-coding regions [34,35]. Indeed, we find that genes with Isw1-dependent shifts are enriched with several histone modifications and depleted of other modifications (Figure 4d-f). Furthermore, modifications that are enriched at genes with Isw1 shifts tend to peak at mid-coding regions, while modifications that are depleted at these genes tend to peak around the TSS. Hence, Isw1-shifts are correlated with histone modifications, both across genes and within genes (Figure 4e).

Combined analysis of these modifications, together with other features (mRNA levels, gene length and cryptic initiation), shows that the most significant effect is from trimethylation of H3K79 (Figure S6 in Additional file 1). This modification peaks at the mid-coding region and is the most strongly correlated with Isw1 shifts, both before and after controlling for the other features. For example, while the average Isw1 shift of +5 nucleosomes is approximately 10 bp over all genes, it is only approximately 1 bp for genes with low H3K79me3 and approximately 17 bp for genes with high levels of this modification (Figure 4d). Other modifications had only minor effects in the combined analysis, although we cannot exclude the possibility that they directly influence Isw1.

### Isw1 remodeling is enriched at cryptic initiation sites

**Figure 5:**
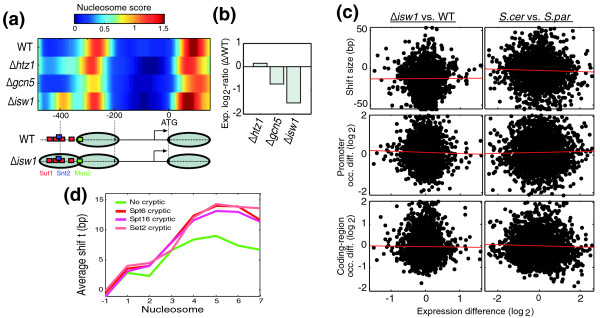
**Isw1 effects on gene expression and cryptic initiation**. **(a) **Nucleosome scores for *TMA10 *show stabilization of the -2 nucleosome in *Δisw1*, which covers multiple binding sites [57]. **(b) **Log_2 _expression ratios (mutant divided by wild type (WT)) for *TMA10 *in the three deletion strains. **(c) **Expression differences are not correlated with Isw1-dependent changes in nucleosome positioning for both comparison of wild-type with *Δisw1 *cells (left) and comparison of the two species (right). Left: scatterplots of log_2 _expression changes in *Δisw1 *versus changes in nucleosome positioning in *Δisw1*. Right: scatterplots of log_2 _expression ratios of the two species versus difference in the effects of *ISW1 *deletion on nucleosomes in the two species. Top: shift size at the +5 nucleosome; in the right panel, minus and plus reflect upstream and downstream shifts, respectively, and in the left panel they reflect larger Isw1 shifts in *S. cerevisiae *and *S. paradoxus*, respectively. Middle: differences in promoter occupancy. In the right panel, log_2_-ratio of the number of reads that map to within 250 bp upstream of the TSS in *Δisw1 *versus wild type. In the left panel, differences in that log_2_-ratio between the two species. Bottom: differences in coding-region occupancy. Same as in the middle panel but for reads that map to the first 500 bp of each coding region. In all cases, red lines represent the linear least square fit, and no significant correlation was observed (*P *> 0.05). (**d**) Average sizes of Isw1-dependent upstream shifts at nucleosomes +1 through +7 for genes with detected cryptic initiation in three mutant strains (for Spt6, Spt16 and Set2) and for genes without cryptic initiation in any of the three mutants. The three datasets of cryptic initiation include 960, 1,130 and 429 genes, respectively, and are all strongly associated with long genes (median length of 2,063, 2,090, and 2,453, compared to 857 for genes without cryptic initiation). Long genes are also enriched with Isw1-dependent shifts (Figure 4d; Figure S6 in Additional file 1).

We next asked whether remodeling by Isw1 influences the regulation of gene expression. To examine the genome-wide correlation between the effects of Isw1 on nucleosome positions and on gene expression, we compared the expression profiles of wild-type and *Δisw1 *strains, as well as *Δhtz1 *and *Δgcn5 *control strains (Figure S7 in Additional file 1). Although *Δisw1 *displayed the most extensive differences in nucleosome positioning, changes in gene expression in this strain were minor, with only approximately 1% of the genes altered by at least 2-fold and approximately 4% of the genes by at least 1.5-fold. At some genes, changes in gene expression correlated with Isw1-dependent nucleosome remodeling. For example, the -2 nucleosome of the *TMA10 *gene is evicted in all strains, except for *Δisw1*, where it covers multiple transcription factor binding sites (Figure 5a). Consistent with this, the expression level of *TMA10 *decreased in *Δisw1 *(Figure 5b).

However, in contrast to *TMA10*, the nucleosome occupancy of most promoter binding sites was not altered by deletion of *ISW1*, as the majority of Isw1-dependent nucleosome changes occur within coding regions. Furthermore, altered gene expression was not enriched at genes whose nucleosome positions or occupancies were affected by *ISW1 *deletion (Figure 5c; Figure S7 in Additional file 1). Similarly, expression differences between the two species were not correlated with species-specific effects of *ISW1 *deletion (Figure 5c; Additional file 3). These results are consistent with recent work that demonstrated that, for the *MET16 *gene, nucleosome remodeling and transcription regulation reflect distinct functions of Isw1 [36]. Similarly, expression changes were only weakly associated with differences in nucleosome positioning for *Δhtz1 *and *Δgcn5*(Figure S7 in Additional file 1).

Thus, changes in nucleosome positioning in *Δisw1 *are generally not associated with regulation of transcription levels, and are highly enriched at mid-coding regions. These results may indicate that Isw1-dependent remodeling is required primarily for maintaining normal chromatin structure at coding-regions during PolII elongation. In the absence of Isw1, coding-region nucleosomes may be perturbed during transcription elongation, resulting in the observed shifts, as well as fuzziness of nucleosome positioning and decreased occupancy. We reasoned that such perturbed chromatin structure may allow aberrant transcription initiation from cryptic sites within coding regions, as previously shown for defects in various elongation factors [37-43]. Consistent with this, we found that coding-regions with Isw1-dependent shifts were enriched with cryptic initiation sites, as mapped in strains with defects in Spt6, Spt16 [37] and Set2 [44] (Figure 5d). This suggests that genes that are prone to defects in chromatin structure that permit cryptic initiation are also more sensitive to deletion of Isw1, linking Isw1 to suppression of cryptic initiation. Indeed, Isw1 was found as one of the 50 factors whose deletion promotes cryptic initiation at the *FLO8 *gene [37].

### Isw1 effects are comparable in magnitude, but do not correlate, with PolII effects

**Figure 6:**
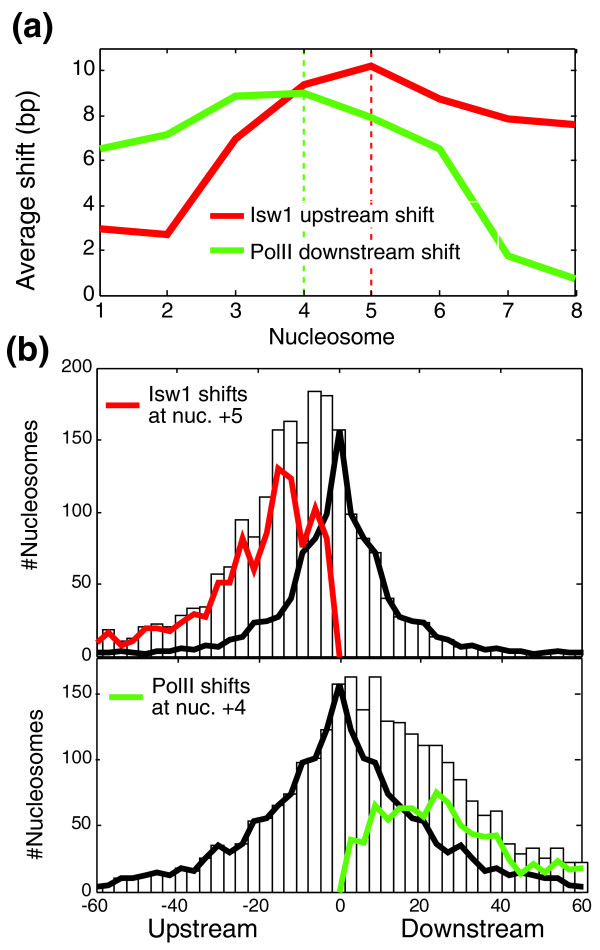
**The extent of shifts in nucleosome positioning is comparable between Isw1 and PolII**. **(a) **Average shift size for nucleosomes +1 through +7 upon deletion of *ISW1 *(red, upstream shifts) or inactivation of PolII (green, downstream shifts). Opposite shifts (downstream for *ISW1 *or upstream for PolII) were given negative values. **(b) **White bars display the distribution of observed shift sizes (positive and negative values reflect upstream and downstream shifts, respectively) for Isw1 (top) and PolII (bottom). Nucleosomes +5 and +4 were chosen in this analysis for Isw1 and PolII, respectively, as these had the most extensive effects. Assuming that Isw1 only shifts nucleosomes upstream, and that the same amount of genes display upstream and downstream shifts due to errors in estimation of nucleosome positioning, we can decompose the observed shifts into those due to errors (black) and those reflecting the activity of Isw1 (red). This analysis predicts that *ISW1 *deletion shifts the +5 nucleosome for 50% of the genes. Similarly, we assumed that PolII only shifts nucleosomes downstream and decomposed the observed shifts into errors (black) and PolII activity (green), with the latter predicted to occur for 33% of the genes. Note that even if we relax these assumptions and simply count the number of observed Isw1 (upstream) shifts and PolII (downstream) shifts, then we obtain a similar fraction of genes (for example, approximately 40% of the genes are shifted by at least 15 bp for both Isw1 and PolII; not shown).

Finally, we compared the nucleosome shifts in *Δisw1 *to the nucleosome shifts found upon inactivation of PolII [45]. Inactivation of PolII shifts nucleosomes downstream of their native positions (towards the 3' end), as opposed to the upstream shifts in *Δisw1*. Thus, some nucleosomes can adopt at least three stable positions: the native position occurring in the wild type; an upstream position when the activity of Isw1 is compromised; and a downstream position when PolII is inactivated. However, although some nucleosomes are shifted both by deletion of Isw1 and inactivation of PolII, we could not detect a consistent association between the two (*r *= -0.02), suggesting that different factors determine the susceptibility of nucleosomes to Isw1 and to PolII. Furthermore, Isw1-dependent shifts are localized to mid-coding regions while PolII shifts are also observed at the 5' ends of coding regions (Figure 6a).

Importantly, the extent of shifts in nucleosome positioning appears to be comparable for Isw1 and PolII, and, if anything, is even larger for Isw1 (limiting the comparison to upstream shifts in *Δisw1 *and downstream shifts for PolII inactivation). First, in both cases approximately 40% of the genes have shifts larger than 15 bp. Second, assuming that nucleosomes are only shifted upstream in *Δisw1 *and therefore that downstream shifts in *Δisw1 *reflect the extent of errors in calling nucleosome positions, we estimate that approximately half of the +5 nucleosomes are shifted upstream in *Δisw1 *(Figure 6b). Similar analysis of PolII inactivation (assuming that nucleosomes are only shifted downstream and that upstream shifts reflect the extent of errors) suggests that only a third of the +4 nucleosomes are shifted downstream (Figure 6b).

## Discussion

Previous studies implicated Isw1 in both transcription initiation (through chromatin modulation at promoters) and transcription elongation (through chromatin modulation at coding regions) [15,29,46]. These studies reached their conclusions based on the analysis of individual genes. Here we analyzed the contribution of Isw1 to the genome-wide nucleosome profile. Our data suggest that the primary remodeling function of Isw1 is at coding regions, with its deletion altering the occupancy, fuzziness and position of a large fraction of the mid-coding nucleosomes.

Some of the changes we observe may reflect indirect effects of *ISW1 *deletion or perhaps be due to technical limitations of our method (for example, the degree of MNase digestion differed a bit between some of the strains; see Figure S1 in Additional file 1). We thus focused most of the analysis on the shifts in nucleosome positions, rather than changes in occupancy. These shifts are most likely to reflect the direct activity of Isw1 for a number of reasons. First, shifts are technically less sensitive to the degree of MNase digestion. Second, the shifted positions of nucleosomes in the *Δisw1 *strain are better explained by sequence-based affinity models than are the wild-type nucleosome positions. Third, nucleosome shifts are consistent with the known catalytic activity of Isw1. Finally, the shifts we observe display distinctive position (mid-coding) and direction (upstream) that are consistent with a role of Isw1 in elongation [15]. Nonetheless, we cannot conclusively distinguish between the direct effects of Isw1 and other indirect effects.

**Figure 7:**
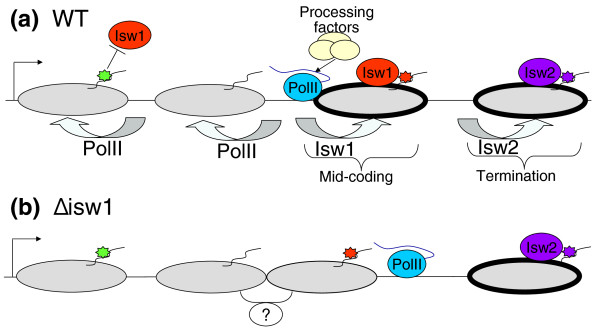
**Model for the nucleosome remodeling function of Isw1**. **(a) **Isw1 is recruited (or activated) by particular histone modifications (red stars; possibly H3K79me3) at mid-coding regions and repelled (or inhibited) by other modifications around the TSS (green stars). Isw1 generates a nucleosomal barrier (illustrated as thick nucleosome edges) that transiently delays PolII and facilitates its interaction with mRNA processing factors. This activity of Isw1 (and/or the presence of a delayed PolII) slides the Isw1-regulated nucleosome downstream towards an energetically less favorable position, which is opposite to the normal effect of PolII on nucleosomes. Isw2 performs a similar function but at the transcription termination (or start) nucleosomes, due to interactions with different factors and histone modifications (purple stars), suggesting a division of labor between Isw1 and Isw2. **(b) **In *Δisw1 *cells, chromatin structure within the coding region is less repressive, thus impairing PolII interaction with processing factors and allowing cryptic initiation. No linker is found between the Isw1-dependent nucleosome at the mid-coding region and its adjacent upstream nucleosome, perhaps indicating that these nucleosomes invade DNA territories occupied by their neighbors [47] or that they are held together by an unknown mechanism. WT, wild type.

The directionality of shifts towards the 5' end of genes, opposite to the direction of transcription elongation and to the shifts found when PolII is inactivated, are consistent with a function of Isw1 in elongation (Figure 7). Indeed, previous work has shown that Isw1 coordinates transcription elongation with mRNA processing and transcription termination [15]. It is tempting to speculate that Isw1 generates a nucleosomal barrier at mid-coding regions that transiently delays PolII and facilitates its interaction with mRNA processing factors (Figure 7). The formation of a nucleosomal barrier, and/or the delayed PolII itself, may cause a directional downstream shift in the positions of the Isw1-regulated nucleosomes, thus accounting for the observed shifts in *Δisw1*.

Which genes are remodeled by Isw1 and how is this specificity maintained? Shifts are enriched at genes with intermediate expression levels, and are generally not associated with particular Gene Ontology annotations, sequences, or DNA-binding factors (Figure S8 and Supplementary Methods in Additional file 1). However, the apparent selectivity of Isw1 to the mid-coding regions of a subset of genes might be explained by histone modifications that are enriched (or depleted) at these regions. These modifications may affect the recruitment or activity of Isw1 [34]. Such a recruitment mechanism is particularly suitable for generating specificity within coding regions, as opposed to promoters, since transcription factor binding sites are generally absent from coding regions. Moreover, recruitment of Isw1 by histone modifications might explain its widespread activity. Consistent with this, H3K79me3 peaks at mid-coding regions and is highly enriched at genes with Isw1 shifts. For example, upstream shifts are found at 14% and 74% of the 1,000 genes with lowest and highest H3K79me3 values, respectively. This strong correlation might indicate a direct association that can explain much of the specificity of Isw1 remodeling.

In addition to histone modifications, Isw1-dependent shifts are enriched at genes where cryptic initiation has been detected in other mutant strains. While the set of genes with cryptic initiation in *Δisw1 *might be different, this association suggests that certain genes are susceptible to defects in chromatin structure during elongation, which leads to cryptic initiation. Such genes may thus be subjected to tight regulation of chromatin structure, which could partially rely on Isw1.

Notably, deletion of *ISW1 *resulted also in significantly shorter inter-nucleosomal linker regions, or even loss of linkers, at the mid-coding region, which is not compatible with the statistical positioning model (Figure 4b). In fact, 43% of the predicted distances between these shifted nucleosome pairs in *Δisw1 *are smaller than 147 bp and 25% are even smaller than 137 bp (compared to approximately 16% smaller than 147 bp and approximately 9% smaller than 137 bp in the wild type or the control strains). While some of these cases may reflect errors in the estimation of nucleosome positions, their high occurrence suggests that many nucleosome pairs are indeed closer than 147 bp to one another. This might be due to neighboring nucleosomes that do not bind to the DNA simultaneously, thus eliminating steric hindrance. However, recent studies have also shown that nucleosomes could in fact invade DNA territories occupied by their neighbors, such that the distance between neighboring nucleosomes is smaller than 147 bp [47]. This phenomenon could be due to partial unwrapping of nucleosomal DNA [48], nucleosome remodeling (by factors other than Isw1) [49], or loss of H2A/H2B dimers [50]. It would thus be interesting to further examine the properties of these adjacent *Δisw1 *nucleosome pairs and their dependence on nucleosome remodeling and transcription elongation.

Our results suggest a 'division of labor' between the homologous factors Isw1 and Isw2 (Figure 7). While Isw2 is involved in maintaining repressive chromatin structure by sliding nucleosomes at the 5' and 3' ends of genes, thus preventing antisense transcription and initiation from suppressed genes, Isw1 may perform a similar function at the mid-coding region. It is thus possible that Isw1 and Isw2 perform similar catalytic functions but at different nucleosomes. This specificity may be linked to their different interacting partners (Ioc2-4 for Isw1 and Itc1 for Isw2) or to direct interactions with different modified histones, such as H3K79me3.

## Conclusions

This work suggests that Isw1 has a widespread influence on the positions of nucleosomes at the mid-coding regions of genes. These effects of Isw1 might be related to a role of Isw1 in transcription elongation and in preventing cryptic initiation within genes. The specificity of Isw1 to mid-coding nucleosomes and the distinct effects of Isw1 and Isw2 may be due to interactions with histone modifications and particularly with H3K79me3.

## Materials and methods

### High-throughput sequencing of mono-nucleosomes from wild-type and mutant strains

Deletion strains were constructed on the background of *S. cerevisiae *(*BY4743*) and *S. paradoxus *(*CBS 432 ho::nat MATα*) using standard techniques, introducing G418 and Kan resistance in *S. cerevisiae *and *S. paradoxus*, respectively. We verified that these deletions did not cause cell-cycle defects (Figure S9 in Additional file 1). Mono-nucleosomal DNA was isolated from cells grown to log-phase in rich media (YPD medium, 30°C) by digestion with MNase (see Supplementary Methods and Figure S1 in Additional file 1 for full details). Mono-nucleosomal DNA from the two species was pooled and subjected to Illumina high-throughput sequencing with one lane for wild-type strains and two lanes (biological repeats) for each of the mutant strains. Similarly, one lane was used to sequence the wild-type hybrid and two lanes for each of the mutant hybrids formed by mating the respective mutants from the two species. Data for biological repeats was averaged.

Reads of 34 to 40 bp were mapped to the genomic sequences of *S. cerevisiae *and *S. paradoxus *with Eland, allowing up to two mismatches within the first 32 bp; approximately 50% of the reads were mapped to a single location in one of the genomes, or were mapped to single locations in both genomes but with at least two more mismatches to one genome. These reads could thus be confidently mapped to a specific location in one of the genomes and the remaining reads were excluded. The genomic similarity between the two yeast species is approximately 85%, with only 13% of the aligned sequences having less than two mismatches for a single read length (36 bp). Thus, our approach of sequencing the two species together excludes approximately 13% of the genome, in which no reads are unambiguously mapped to either species, but does not affect the majority of the genome. Since we look for differences between wild-type and mutant strains, and use the same methods for mapping reads in both cases, this approach should have no effect on the observed differences but only hinders the detection of differences at highly conserved regions, which are excluded from the analysis.

### Processing of mono-nucleosome sequencing data

Since reads of approximately 36 bp corresponded to the ends of approximately 150-bp fragments, the location of each mapped read was converted into the expected center position of the original DNA fragment. This was done by assuming a constant fragment length for each lane and each species. This length was estimated as the median distance between peaks of read-density in the forward strand and consecutive peaks of reads from the reverse strand (Table S1 in Additional file 1).

We obtained the number of reads that mapped to each base pair and transformed it to 'nucleosome occupancy', that is, the number of reads that cover each base pair, assuming that reads correspond to mono-nucleosome fragments of 147 bp. For prediction of center nucleosome positions we also defined 'nucleosome scores' by Gaussian filtering of the number of reads at each base pair, with a window of 50 bp and standard deviation of 25 bp [19]. This transformation produces sharper peaks and allows a better estimation of nucleosome center positions. We estimated the positions of nucleosomes as peaks of nucleosome scores that were (i) not among the 10% peaks with lowest scores, and (ii) not within 100 bp of another peak with higher score. The number of nucleosomes defined by these criteria corresponded to approximately 80% of nucleosomal DNA, as estimated by previous studies [24].

For comparative analyses, nucleosome scores from all samples were normalized to the same distribution using percentile normalization. The raw data of mapped reads and the normalized nucleosome scores are available at the Sequence Read Archive and Gene Expression Omnibus databases (accession number GSE18939).

### Comparison of nucleosome positioning

We compared nucleosome positioning at genes and promoters (1 kb) for each gene between different strains. If two nucleosomes from one strain paired with the same nucleosome from the other strain, then the one that is more distant from the single nucleosome was regarded as a possible nucleosome loss/gain. Nucleosomes whose positions differed by at least 15 bp between strains and that had a *t*-test *P*-value lower than 0.05 were regarded as a possible nucleosome shift. The *t*-test was performed by comparing the distribution of read positions of the two strains around the center positions of the respective nucleosome (taking all reads that map to at most 30 bp from the center position of one of the strains). Nucleosomes whose occupancy level differed by at least two-fold (after correcting for the overall difference in occupancy levels between the corresponding samples) were regarded as a possible occupancy change.

Each potential nucleosome loss/gain was also required to have at least two-fold higher occupancy in the strain with the nucleosome (compared with the strain without the nucleosome) and that this nucleosome will be supported by at least eight reads. To further increase the confidence of the predicted nucleosomal changes, we repeated the analysis above only for the reads that mapped to the forward strand and (separately) only for the reads that mapped to the reverse strand. We required that potential changes would pass all of the above thresholds in each of the strands, and that nucleosomes at positions of potential changes are mapped in the forward and reverse analyses to within 30 bp of their positions in the combined analysis (10 bp for shifts).

### Bound versus unbound genes

We defined Htz1 bound and unbound genes as the highest 20% and lowest 40% ChIP ratios, respectively [27]; Gcn5 bound and unbound genes were defined as those with *P*-values lower than 0.05 and higher than 0.4, respectively. Isw1, Ioc2 and Ioc3 bound genes were defined as in Venters *et al*. [26] and non-bound genes were defined as those that were identified as bound by at least one other factor but not by these particular factors.

### Comparison of expression levels

Genome-wide expression levels of the wild-type and mutant strains were measured for the two species, with a multi-species array, as described previously [51]. Differential expression was defined as at least 1.5-fold differences, although the use of other thresholds did not significantly alter the results (not shown).

## Abbreviations

bp: base pair; ChIP: chromatin immunoprecipitation; H3K79me3: trimethylation of lysine 79 of histone H3; PolII: RNA polymerase II; TSS: transcription start site.

## Authors' contributions

IT performed all analysis of the data and wrote the manuscript. NS carried out all experiments. NB participated in the analysis and wrote the manuscript. All authors conceived and designed the study. All authors read and approved the final manuscript.

## Supplementary Material

Additional file 1 Supplementary methods, Figures S1 to S9 and Tables S1. Click here for file

Additional file 2 Center coordinates of wild-type *S. cerevisiae *nucleosomes, and their shifts in position and changes in occupancy in *Δisw1*. Click here for file

Additional file 3 Lists of genes with conserved, *S. cerevisiae*-specific and *S. paradoxus*-specific shifts in the positions of mid-coding nucleosomes. Click here for file
