# Supplementary material for: Widespread remodeling of mid-coding sequence nucleosomes by Isw1
Source: Genome Biol. 2010 May 10;11(5):R49. doi: 10.1186/gb-2010-11-5-r49 (PMC2898075; doi:10.1186/gb-2010-11-5-r49)
Supplement: Additional file 1 — Supplementary methods, Figures S1 to S9 and Tables S1. [file gb-2010-11-5-r49-S1.PDF]

## Supplementary Methods

### MNase digestion and sample preparation for high throughput sequencing

Cells were grown to $OD_{600}$ of 0.8-1.0 at 30°C from overnight starter diluted to 100 ml of YPD medium at $OD_{600}$ of 0.15. Formaldehyde solution (37%) was added to final concentration of 1%, followed by shaking at 30 °C for 15 min. The formaldehyde was quenched by adding glycine to 0.125 M and letting to stand at room temperature for 5 min. Cells were collected by centrifugation at 3000 g for 5 min and washed twice with same volume of ice-cold water. At this point pellet can be stored at -80°C. Cells were resuspended in 10 ml Buffer Z (1M sorbitol, 50 mM Tris-HCl pH = 7.5, freshly added 10 mM β- mercaptoethanol), 70 μl of zymolase was added and the cells were incubated at 30°C for 30 min. Then the spheroplasts were collected by centrifugation at 3000g for 10 min, washed with 10 ml Buffer Z and resuspended in 0.5 ml Buffer NP (1M sorbitol, 10 mM Tris- HCl pH = 7.5, 50 mM NaCl, 5 mM MgCl2, 1 mM CaCl2, freshly added 1 mM β- mercaptoethanol, NP-40 0.075%, 0.5 mM spermidine). Then the sample was split for 2 aliquots, digested for 20 min at 37°C with 0.5 and 2 units of MNase. The reactions were stopped by addition of 0.1 ml of 5% SDS with 50 mM EDTA to each reaction. Cross-linking was reversed at 65°C for 4 h, samples were diluted with 0.4 ml water and 5 μl of RNase A (10 mg/ml , Sigma) were added. The samples were incubated at 37°C for 1 h, then 5 μl of Proteinase K (20 mg/ml) were added to each sample and were incubated at 65°C for 1 h. The DNA was extracted by phenol-chloroform-isoamyl alcohol (PCI), washed with chloroform-isoamyl alcohol (CI) and precipitated with ethanol. The digestion products were resolved on a 2% agarose gel, and mononucleosomal size band (~150 bp) was isolated by Wizard SV gel clean-up kit (Promega). An additional ethanol precipitation was performed to improve purity of the sample and to concentrate the input DNA.

MNase digestion gels are shown in Figure S1, and the average offset between peaks of read-density from the forward and backward strands are given in Table 1 (these reflect the typical length of the sequenced DNA fragments and thus the degree of MNase digestion). These data show that all of our samples are relatively "under-digested" with respect to Weiner et al., with slight differences between samples. For example, the average strand-offsets are between 135 and 160 for all samples, with a difference of ~5-10bp between wild-type and Δisw1 samples and a similar difference between wild-type and the other mutant strains. These differences may have some influence on our data. However, the overall differences that we report for Δisw1 (reduced occupancy, increased fuzziness and upstream shifts of coding-region nucleosomes) are consistent among the two species and the hybrid and are not observed for the two other mutants, while the differences in the degree of digestion are similar for the three mutant strains. It is therefore unlikely that these patterns are due to differences in under-digestion.

### Library preparation and sequencing

The similar amounts of DNA from three independent biological repeats were mixed and diluted to 1 ng/ml concentration. The ChIP-Seq DNA Sample Prep Kit (IP-102-1001) and the standard protocol by Illumina were used to create libraries for sequencing, with the modification: the fragments with ligated adaptors were subjected directly to amplification, skipping the gel purification and size selection step. All

other steps of cluster formation and sequencing procedure were performed following standard protocols for Illumina GA2 instrument.

**Analysis of genes containing Isw1-dependent shifts**

GO analysis showed strongest enrichment for intracellular transport ($P=10^{-6}$, before correction for multiple hypothesis) and weaker enrichment for related terms. MEME was unable to identify motifs that are enriched at positions of ISW1-shifted +5 nucleosomes compared to non-shifted +5 nucleosomes, and analysis of k-mer frequencies at these regions also did not identify significant differences (**Fig. S8**). Only weak enrichments (less than 2-fold or non-significant) were identified for Isw1-dependent shifts at targets of various DNA-binding factors. Isw1-dependent shifts were underrepresented at TATA-containing genes (14% of the shifts at TATA-containing genes, compared to ~20% of all genes).

**Supplementary Table 1.** Average offset between consecutive peaks of read density in the Forward and Reverse strands.

| | **S.cerevisiae** | **S.paradoxus** | **Hybrid** |
|---|---|---|---|
| **Wild-type** | 145 | 147 | 147 |
| **Δisw1** | 150,152 | 155,160 | 150,155 |
| **Δhtz1** | 147,150 | 155,160 | 155,160 |
| **Δgcn5** | 135,140 | 150,152 | 150,152 |

## Supplementary Figures

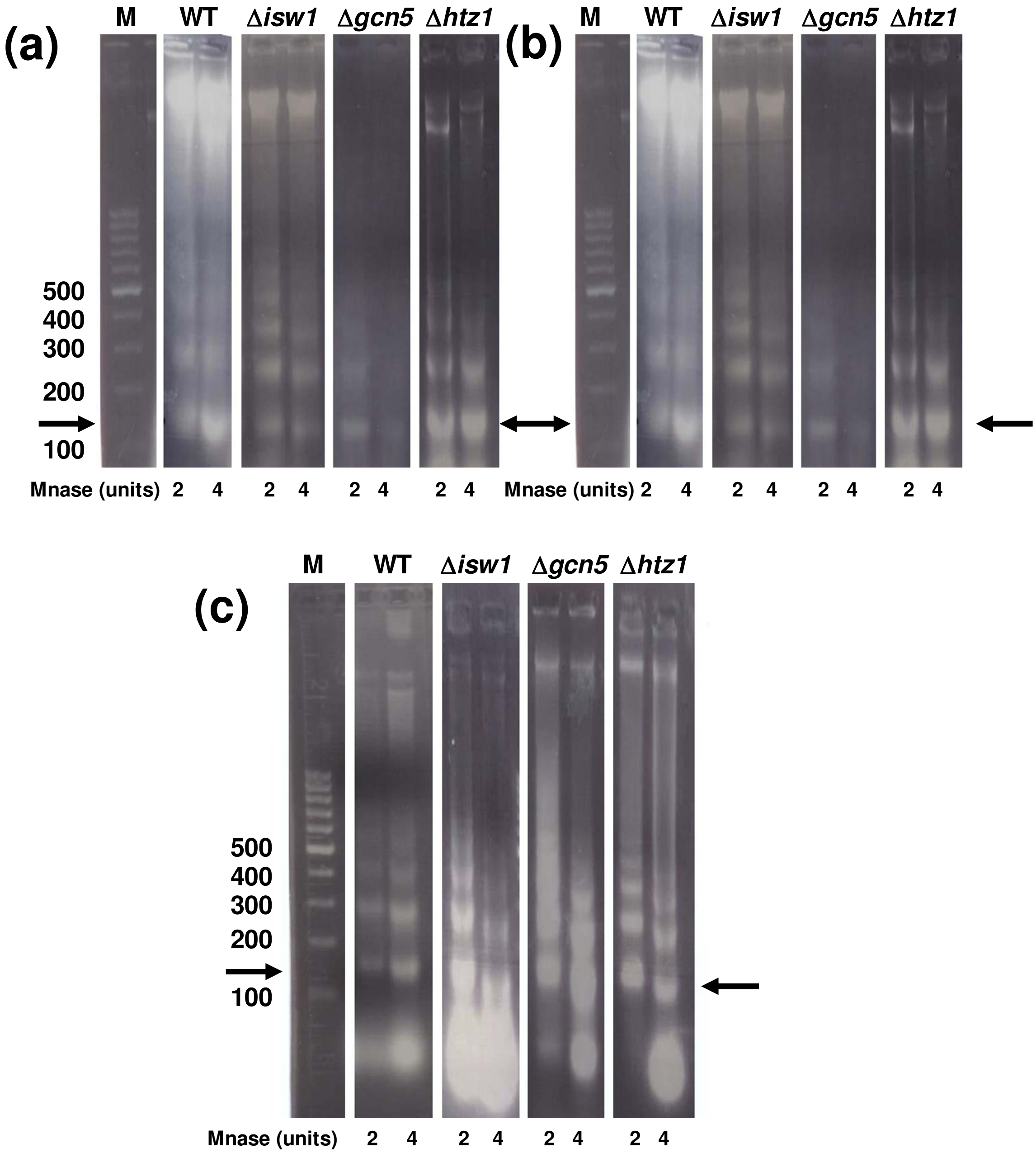


**Figure S1. Gel electrophoresis analysis of Mnase digestion.** DNA isolated from the WT or the indicated deletion mutants was digested using two Mnase concentrations (2 and 4 units) and analyzed in 2% agarose gel. Mononucleosomal band from both reactions (indicated by arrows) was isolated and subjected to high throughput sequencing. Typical digestion patterns of DNA are presented for all strains of *S. cerevisiae* (a), *S. paradoxus* (b) and their hybrid (c

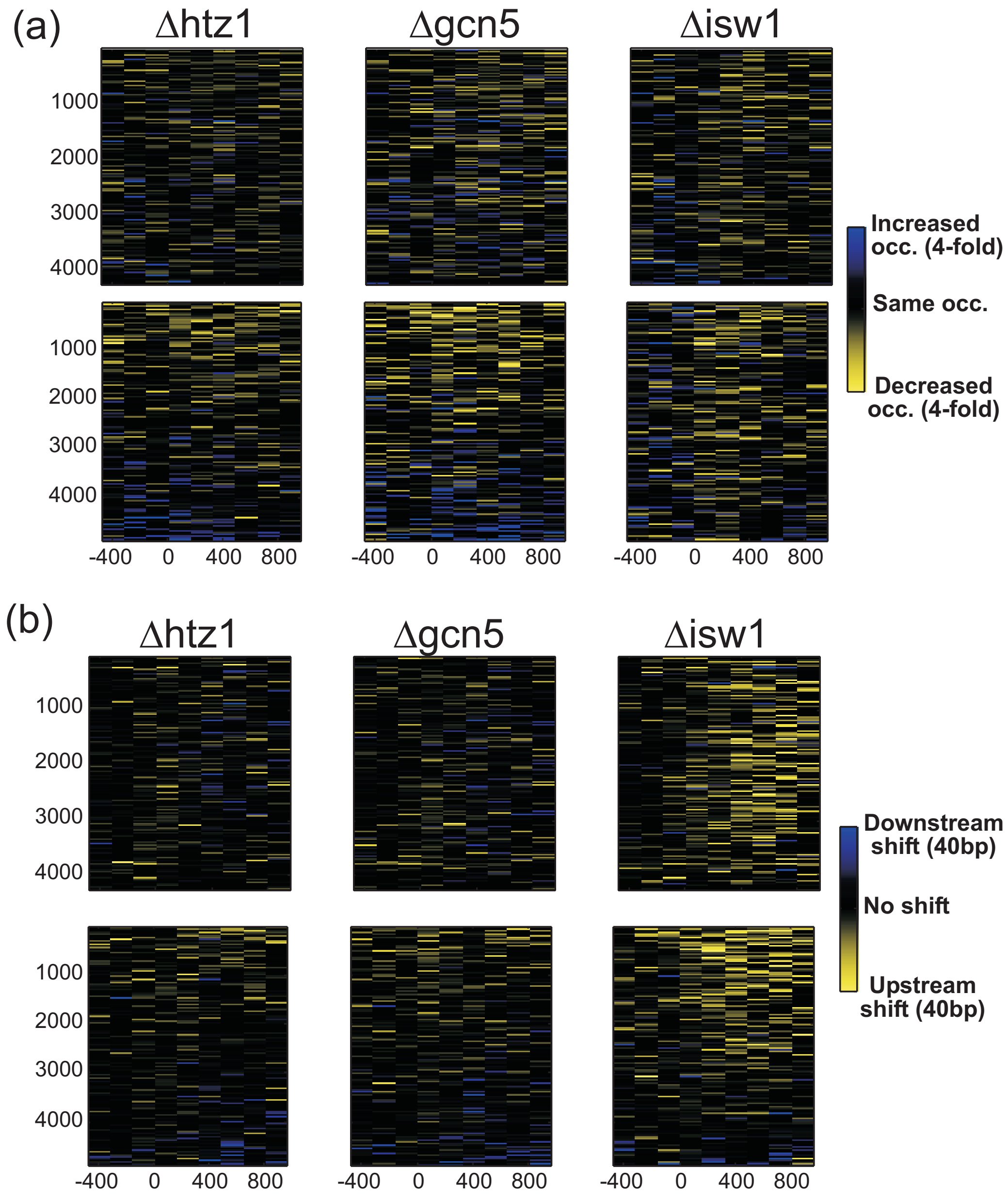


**Figure S2. Heatmaps of changes in nucleosome occupancy (a) or positions (b) for the three mutant *S. cerevisiae* strains (relative to the wild-type).** For each wild-type nucleosome, we examined the changes in occupancy and position. Genes were sorted either by transcription rates [54] (top panels of a,b), or by the sum of changes across each gene (bottom panels of a,b). This data is also provided in Supplementary table 1.

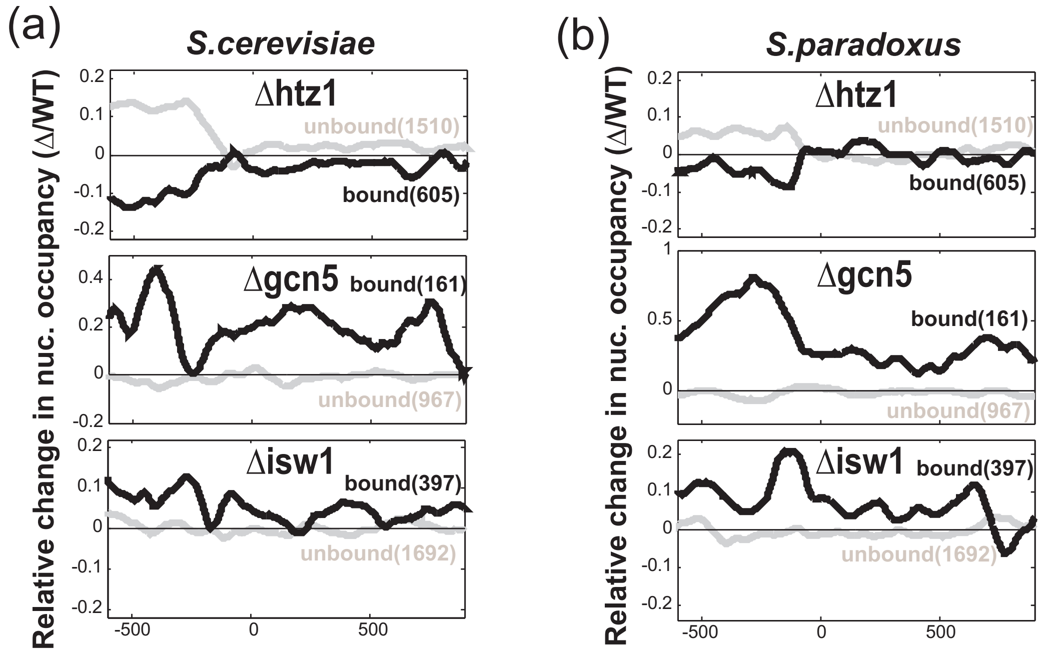


**Figure S3. Comparison of the mutant effects on bound and unbound genes.** For each position (relative to start codon) the distribution of nucleosome occupancies of all genes in the mutant strain was normalized to that of the wild-type. The ratio of normalized occupancies (Δ/WT), which therefore has an average of zero, was compared between genes whose promoters was identified as bound (black curve) or unbound (grey curve). Curves were smoothed with a sliding window of 50 bp, and the number of bound/unbound genes is indicated in parentheses. The difference between bound and unbound genes was smallest for Isw1 (note also the different scales of the y-axis). Similar results were obtained for the Ioc2-3 (not shown) and between *S. cerevisiae* (a) and *S. paradoxus* (b).

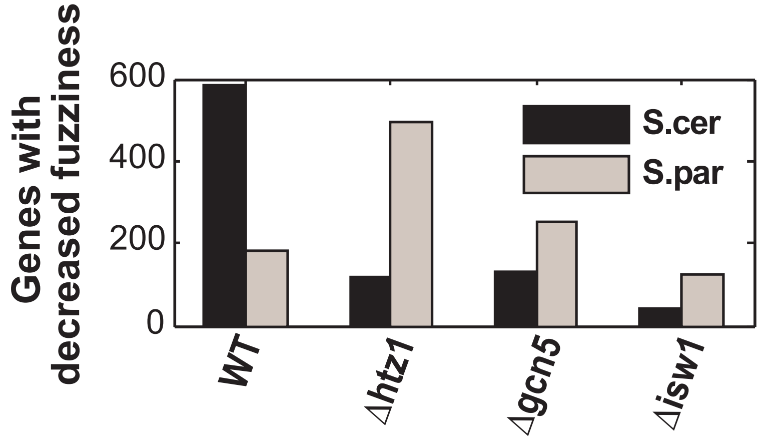


**Figure S4. Number of genes with decreased fuzziness at each strain for *S. cerevisiae* (black) and *S. paradoxus* (grey).** Genes were defined to have decreased fuzziness in a particular strain if the percentage of reads that map to within 20bps of the estimated nucleosome center positions was higher by at least 5% than that of all other strains.

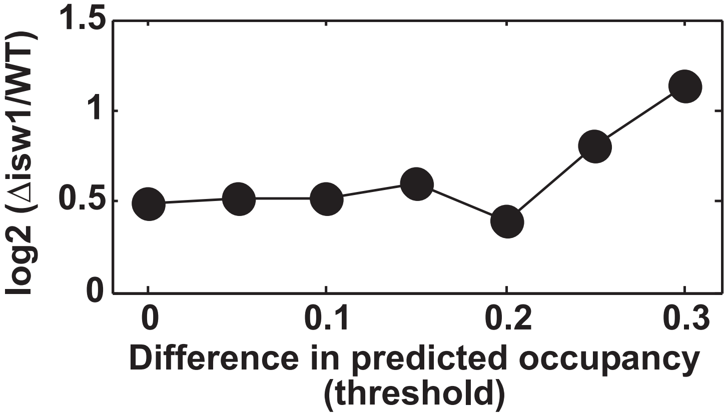


**Figure S5. Isw1-shifted nucleosomes are usually shifted to positions which are more consistent with sequence-based predictions of nucleosome positions.** For each Isw1-shifted nucleosome we compared the predicted nucleosome occupancy at the wild-type position and at the Δisw1 position (taken from from Kaplan et al., with predicted occupancy values between zero and one). We then compared the number of nucleosomes with higher and lower predicted occupancy at Δisw1, with various thresholds for the difference in occupancy. At all thresholds, we find that more nucleosome have increased predicted occupancies than those with decreased predicted occupancies (shown are the log2 of the ratios between the number of nucleosomes with increased vs. decreased predicted occupancies). These enrichments are significant for all threholds ($p$<0.05).

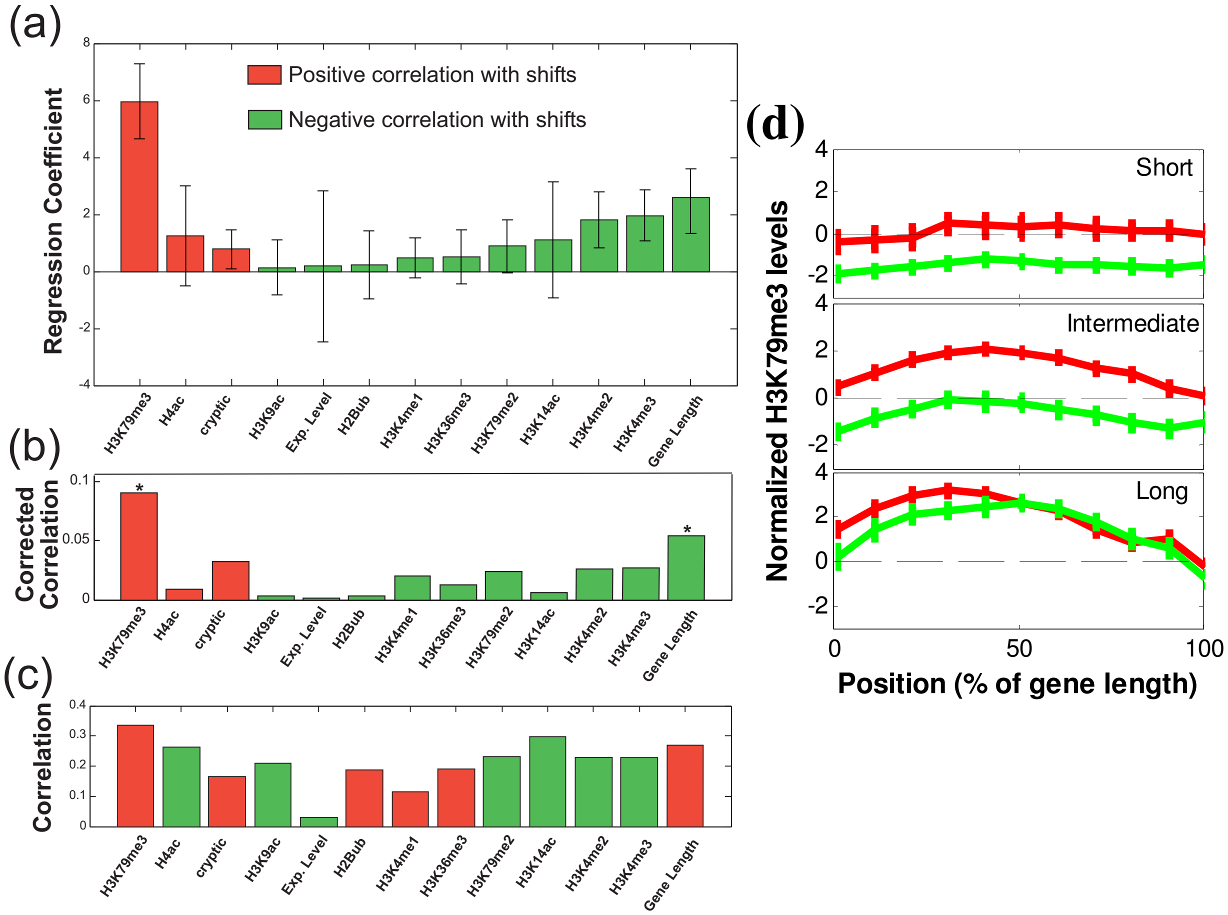


**Figure S6. Combined analysis of multiple predictors of Isw1-dependent shifts.**

**(a)** We performed multiple linear regression for all factors against the shift of the +5 nucleosome at *Δisw1* as the response variable. Cryptic initiation was quantified as the number of datasets in which a gene was identified as having cryptic initiation. Expression levels and gene-length were $\log_2$-transformed, and all factors were normalized to mean of zero and standard deviation of one. This analysis assigned weights (regression coefficient) for each factor in predicting Isw1-shifts, which are presented as bars. Red and green represent positive and negative weights, and errorbars represent 95% confidence intervals.

**(b)** We examined the correlation of each factor with the residual of Isw1-shifts from a multiple regression analysis with all other factors. This analysis examines whether each factor has an effect on Isw1-shifts which cannot be accounted by combinations of the other factors. Red and green bars represent positive and negative correlations, and asterisks represent significant (P<0.01) correlations.

**(c)** Correlation of each factor with Isw1-shifts (without controlling for the other factors).

**(d)** Although gene-length is positively correlated with Isw1-shifts, both of the combined analyses (a,b) assign *negative* weights to gene-length. Consistent with this, the difference in H3K79me3 between shifted and non-shifted genes is very significant for short (top panel) and intermediate-length genes (middle panel) but much lower for long genes (bottom panel). Red curve represents the average levels of H3K79me3 at genes with Isw1-shifts, and green curve represents the average for genes without Isw1-shifts; errorbars were calculated by bootstrapping. These results may suggest that gene-length is not directly associated with Isw1 shifts but that it is correlated with other factors that are linked to Isw1-shifts. These factors may include histone modifications which are correlated with gene-length (e.g. H3K79me3) as well as cryptic initiation which is also correlated with gene-length. Taken together, it seems more likely that Isw1-shifts are directly linked to histone modifications and to cryptic initiation and that the association with gene length is indirect.

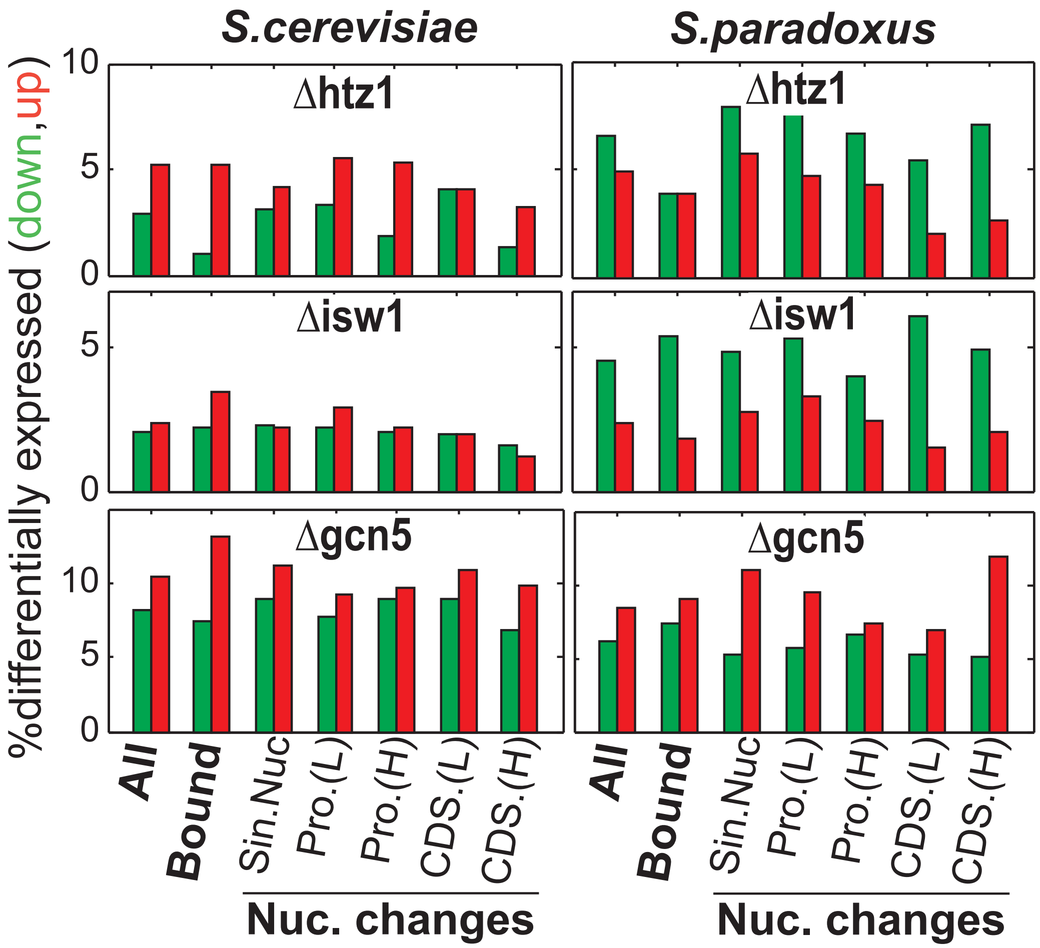


**Figure S7. Percentage of genes with above 1.5-fold differential expression at several sets of genes, for each of the mutant strains and for the two species (green and red represent down- and up-regulation in the mutant strains, respectively).** The sets of genes include all genes (All), genes whose promoters are bound by the respective factor (Bound), and four sets with differential nucleosome positioning at the respective mutant strain: genes with single-nucleosome effects (Sin.Nuc.), genes with at least 1.5-fold lower or higher overall promoter occupancy compared with the wild-type (Pro. (L) and Pro. (H), respectively), and genes with at least 1.5-fold lower or higher overall coding-region occupancy, compared with the wild-type (CDS. (L) and CDS. (H), respectively). The percentage of differentially expressed genes was generally similar in all these gene sets to the genomic average (All), suggesting that differential expression is only weakly associated with differential nucleosome positioning.

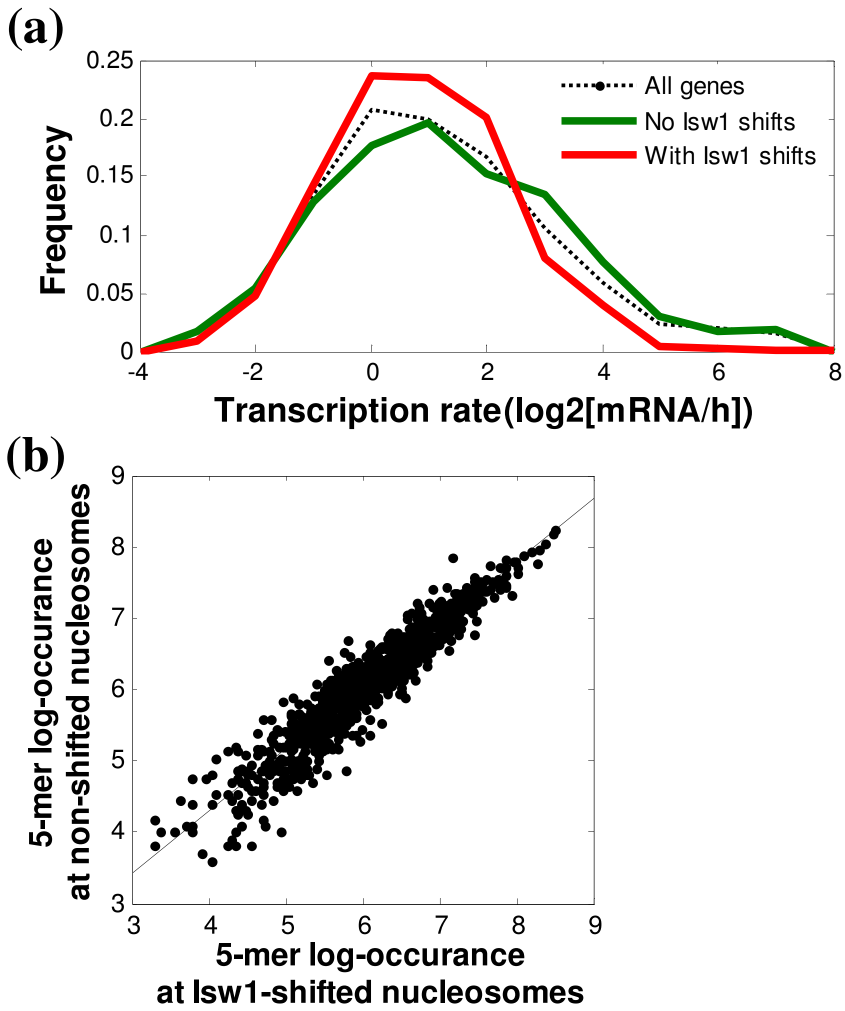


**Figure S8. Analysis of genes containing Isw1-dependent shifts.** **(a)** Distributions of transcription rates [54] for all genes (black), genes without Isw1-dependent shifts at the mid-coding region (green), and genes with Isw1-dependent shifts at the mid-coding region (red). **(b)** log2 of the occurance of each 5-mers at the 200bp around +5 nucleosomes with (x-axis) or without (y-axis) Isw1-dependent shifts.

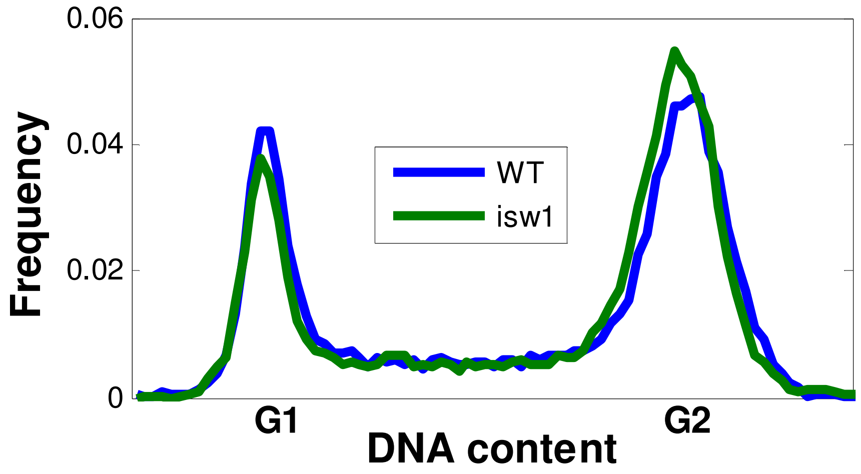


**Figure S9. Similar FACS profiles for wild-type and Δisw1 mutants**. DNA content was measured in wild-type and Δisw1 mutants using SYBR green.
